# Blood Levels of Oxidant/Antioxidant Parameters in Rats Infected with* Toxoplasma gondii*

**DOI:** 10.1155/2016/8045969

**Published:** 2016-09-26

**Authors:** Somayeh Bahrami, Ali Shahriari, Mehdi Tavalla, Somayeh Azadmanesh, Hossein Hamidinejat

**Affiliations:** ^1^Department of Parasitology, Faculty of Veterinary Medicine, Shahid Chamran University of Ahvaz, Ahvaz, Iran; ^2^Department of Biochemistry, Faculty of Veterinary Medicine, Shahid Chamran University of Ahvaz, Ahvaz, Iran; ^3^Department of Medical Parasitology, Faculty of Medicine, Jundishapur University of Medical Sciences, Ahvaz, Iran

## Abstract

Toxoplasmosis is a common parasitic infection in the world. Since increased free radicals and oxidative stress are reported in many parasitic diseases the purpose of the present study was to evaluate the oxidative stress in acute and chronic toxoplasmosis. RH strains of* Toxoplasma* tachyzoites were used in the present study. Twenty-five female rats were infected with the parasite while 25 other rats were as the control group that received normal saline. Zero-, 5-, 7-, 10-, and 45-day postinfection (DPI) blood samples were taken. Some parameters related to oxidant and antioxidants such as antioxidant enzymes, malondialdehyde, and total antioxidant capacity were measured. On day 7 after infection, GPX activity and GSH level were significantly increased and in the mentioned day the amount of total antioxidant capacity was significantly reduced. In other cases, there were no significant differences between the groups in different days. Overall, based on the results it seems that, on day 7 after infection, in infected rats responses to oxidative stress were triggered and led to decrease of total antioxidant capacity. Furthermore, glutathione was increased to cope with stress. It seems that probably antioxidant defense system entered the infection to the chronic phase and changed the parasites stage.

## 1. Introduction

*Toxoplasma gondii* is an obligate intracellular protozoan parasite responsible for toxoplasmosis, a disease that affects many mammals including man [1, 2]. Infection of an immunocompetent individual is usually asymptomatic; however, infection of immunocompromised individuals or congenital infection of a fetus can lead to debilitating or life-threatening illness. Infection generally occurs through either ingestion of oocysts shed in the faeces of a cat, which is the definitive host, or ingestion of viable tissue-cysts in undercooked meat. When primary infection occurs during pregnancy, congenital transmission could occur. Initial infection and acute disease are characterized by the presence of fast-replicating tachyzoites. Around 10–14 days after infection, tachyzoites differentiate into bradyzoites that replicate more slowly and form cysts in tissues throughout the body. Tissue cysts are long-lived and not associated with disease. However, in people with immunodeficiency, such as AIDS or malignancies, rupture of tissue cysts and the transformation of bradyzoites to tachyzoites result in disease reactivation. Congenitally infected individuals are also at risk of repeated disease reactivation, most notably in their brain and eyes, although the reason(s) for this has not been established. Recent investigations indicate that parasitic infections with high tolerance of the host are the result of defense mechanisms which include enhanced generation of reactive oxygen species (ROS) [3, 4]. Cells and biological fluids from an antioxidant defense system aid at suppression of ROS generation and prevention of ROS reactions with cellular components. A balance between oxidants and antioxidant is known to exist under physiological conditions. However, even small changes in oxidant or/and antioxidant levels may disturb its balance and leads to oxidative stress. This situation becomes dangerous when the antioxidant system is unable to prevent oxidative reactions triggered by ROS and directed oxidative modification of lipids, proteins, and DNA [5–7]. Free radicals are produced continuously by normal metabolic processes, but their rate of production increases during certain parasitic infections.

Stage conversion between tachyzoite and bradyzoite forms of* Toxoplasma* is associated with morphological and molecular biological changes, including stage-specific antigen expression and alterations to metabolism [8]. Different studies confirmed that alkaline medium, heat shock protein, and acid conditions can induce stage conversion* in vitro*. These methods appear to rely on stressing the parasites. Having these facts in mind, the present study was designed to compare the antioxidant profile of rats during acute and chronic toxoplasmosis and investigate probable role of oxidative stress in* Toxoplasma gondii* tachyzoite-bradyzoite interconversion.

## 2. Materials and Methods

### 2.1. Parasite

The virulent RH strain of* T. gondii* was obtained from Jundishapur University of Medical Sciences, Ahvaz, Iran. Tachyzoites of this strain were collected by serial intraperitoneal passages in BALB/c mice. Parasites (1 × 10^5^) were inoculated in the mice, and after 72 hours, tachyzoites were provided by repeated flushing of the peritoneal cavity by phosphate buffered saline (PBS). Tachyzoites were then harvested and centrifuged at 200 ×g for 5 min at room temperature to remove peritoneal cells and cellular debris. The supernatants were collected and centrifuged at 800 ×g for 10 min. The pellets, enriched with parasite tachyzoites, were recovered with PBS and used in the experiments [9].

### 2.2. *In Vivo* Study

Fifty female Wistar rats, 8 to 10 weeks old, approximately 250–280 g, were purchased from laboratory animal breeding council (Jundishapur University of Medical Science, Ahvaz, Iran). They were housed in a room under controlled temperature (24 ± 2°C), lighting (12 h light/dark cycle), and relative humidity 40–70% conditions. During experiment, all rats had free access to water and standard rat chow. After 1 week acclimation, firstly modified agglutination test was used to ensure rats were not already infected with* Toxoplasma*, and then they were divided randomly into two equal groups: The infected and control groups were each inoculated intraperitoneally with 1 × 10^7^ organisms [10] and normal saline, respectively. On days 0, 5, 7, 10, and 45 after infection, five rats from each group were euthanized in a glass desiccator jar for open-drop anesthesia with chloroform following standard animal ethics guidelines of Iran. Blood samples were obtained by cardiac puncture into sterile vacuum tubes with and without anticoagulant (EDTA). Serum was separated by centrifuge and stored at −20°C until use. During the present study, the animals were handled according to the recommendation of the Ethics Committee, Shahid Chamran University of Ahvaz, Ahvaz, Iran (Ethical Approval number 29/6/94, 30 Sept. 2014). Acute and chronic toxoplasmosis of was confirmed by PCR and modified agglutination test (MAT), respectively.

### 2.3. DNA Extraction and Polymerase Chain Reaction (PCR) Amplification

Induction of acute toxoplasmosis was confirmed with PCR in infected rats on days 5, 7, and 10 after infection. DNA was extracted from whole blood using a genomic DNA purification kit (CinnaGen, Iran). For detection of* T. gondii* DNA, primers targeting the G529 gene were selected from the literature [11]. PCR reactions included a negative control, consisting of the reaction mix and 2 *μ*L of DNase/RNase-free water and a positive control that consisted of a DNA sample from the tachyzoites of* T. gondii*. All PCR were performed in a 25 *μ*L reaction containing 12.5 *μ*L Taq DNA polymerase master mix Red (Amplicon, Denmark), 1 *μ*M primers, and 50 ng DNA templates. PCR cycling included an initial denaturation at 94°C for 4 min, followed by 30 cycles of denaturation at 94°C for 50 s, annealing at 57°C for 50 s, and extension at 72°C for 60 s. This was followed by a final extension at 72°C for 5 min. PCR products were electrophoresed in 1.5% agarose (SinaClon Bioscience, Iran) in Tris-acetic acid-EDTA (TAE) buffer, stained with Green Safe stain (SinaClon Bioscience, Iran), and visualized under ultraviolet light. Positive samples showed a band of approximately 400 bp.

### 2.4. Modified Agglutination Test

To ensure that chronic toxoplasmosis occurred in rats on day 45 after infection, MAT was used as described by Desmonts and Remington and Dubey and Desmonts [12, 13]. The sera were diluted twofold (1 : 20 to 1 : 320) with phosphate buffered saline containing 0.2 M 2-mercaptoethanol and 50 *μ*L of each dilution was put in a well of 96 U-bottom ELISA plates. Thereafter, 50 *μ*L of the whole formalin-preserved* T. gondii* tachyzoites was added to each serum dilution. The wells were then mixed thoroughly by pipetting up and down several times, covered, and then incubated at 37°C overnight. The test was considered positive when a layer of agglutinated parasites was formed in wells at dilutions of 1 : 20 or higher. Positive and negative controls were included in each test.

### 2.5. Oxidant/Antioxidant Assessment

The determination of the SOD activity was based on the generation of superoxide radicals produced by xanthine and xanthine oxidase, which react with 2-(4-iodophenyl)-3-(4-nitrophenol)-5-phenyltetrazolium chloride to form a red formazon dye. Briefly, 300 *μ*L of mixed substrate was added to 200 *μ*L of diluted hemolysates. The samples were mixed well and 75 *μ*L xanthine oxidase was added to reactions. The absorbance was measured at 505 nm and the SOD activity was then calculated according to the manufacturer's instruction (Ransod®-Randox Lab, Antrim, UK) and expressed as U/mL.

Glutathione peroxidase activity was determined based on the fact that GPX catalyzed the oxidation of glutathione by cumene hydroperoxide. In presence of the glutathione reductase and nicotinamide adenine dinucleotide phosphate (NADPH), the oxidized glutathione was immediately converted to the reduced form with concomitant oxidation of NADPH to NADP^+^. To evaluate GPX activity in hemolysates 10 *μ*L of samples was mixed with 500 *μ*L reagent R1 and 20 *μ*L cumene R2. The absorbance was measured at 340 nm and the GPX activity was then calculated according to the manufacturer's instruction (Ransel®-Randox Lab, Antrim, UK). The enzymes activities were expressed as U/mL.

The procedure to estimate the reduced glutathione (GSH) level followed the method described by Ellman [14]. In this method thiols react with Ellman's reagent (5,5′-dithiobis-(2-nitrobenzoic acid) or DTNB), cleaving the disulfide bond to give 2-nitro-5-thiobenzoate (TNB^−^), which ionizes to the TNB^2−^ dianion in water at neutral and alkaline pH.

To evaluate GSH level in samples, 15 *μ*L of hemolysates was mixed with 260 *μ*L assay buffer (0.1 M sodium phosphate and 1 mM EDTA, pH: 8) and 5 *μ*L Ellman reagents. Samples were incubated for 15 min at room temperature and the TNB^2−^ formation was quantified in a spectrophotometer by measuring the absorbance of visible light at 412 nm. Absorbance values were compared with a standard curve generated from standard curve from known GSH.

Catalase activity was determined spectrophotometrically by the method of Koroliuk et al. [15]. Briefly, 10 *μ*L of sample was incubated with 100 *μ*mol/mL of H_2_O_2_ in 0.05 mmol/L Tris-HCl buffer pH = 7 for 10 min. The reaction was terminated by rapidly adding 50 *μ*L of 4% ammonium molybdate. Yellow complex of ammonium molybdate and H_2_O_2_ was measured at 410 nm. One unit of catalase activity was defined as the amount of enzyme required to decompose 1 *μ*mol H_2_O_2_ per min.

Total antioxidant capacity of serum was measured according to the method of Benzie and Strain [16]. Briefly, a working solution of FRAP (ferric reducing antioxidant power) was provided by mixing buffer acetate with TPTZ solution in HCl. After that FeCl_3_ was added and mixed. 8 *μ*L of serum and 240 *μ*L of mentioned working solution were mixed and incubated for 10 min at room temperature. The optical density of samples was measured at 532 nm. Total antioxidant capacity was expressed as mmol/L.

Malondialdehyde levels in samples were measured using the thiobarbituric acid reaction method of Placer et al. [17]. Quantification of the thiobarbituric acid reactive substances was determined at 532 nm by comparing the absorption to the standard curve of MDA equivalents generated by acid-catalyzed hydrolysis of 1,1,3,3-tetramethoxypropane. To measure MDA level, a working solution containing 15% trichloroacetic acid, 0.375% thiobarbituric acid, and 0.25 N hydrochloric acid was prepared. For each sample, 250 *μ*L serum and 500 *μ*L working solution were mixed and placed in boiling water for 10 min. After cooling the samples were centrifuged at 3000 rpm for 10 min. Finally 200 *μ*L of each supernatant was transferred to microplates and the optical density of samples was measured at 535 nm. The values of MDA were expressed as *μ*mol/L.

### 2.6. Statistical Analysis

The mean value and standard error were calculated for each group of measurements. The Kolmogorov-Smirnov test was used to test the normal distribution of the data before statistical analysis was performed. Statistical analyses were conducted with the general linear model procedure of SAS for Windows version 9.1 (SAS, 1998) to determine if variables differed between groups. Whenever significant differences were found, mean values were compared by the Tukey test. A probability value of less than 0.05 was considered significant.

## 3. Results

After induction of experimental toxoplasmosis, acute toxoplasmosis was confirmed by PCR. In all the samples obtained 5 and 7 days after infection, DNA of* Toxoplasma* was detected in blood. Ten days after infection except for the two samples others were positive. Although in two samples DNA was not detected they were serologically positive. The accuracy of experimental chronic toxoplasmosis induction (samples obtained 45 days after infection) was serologically checked out. In all the samples complete carpet of agglutinated organisms was seen and considered as positive.

The antioxidant enzymes activities, total antioxidant capacity, and MDA level in blood of experimentally infected rats were compared to those of noninfected.

Changes of SOD activity in different days in infected (*p* = 0.91) and uninfected group (*p* = 0.62) were not significant. Also after comparing SOD activity in infected and uninfected groups on the days 0 (*p* = 0.81), 5 (*p* = 0.36), 7 (*p* = 0.75), 10 (*p* = 0.35), and 45 (*p* = 0.80) after infection, significant changes were not found (Table 1).

According to our data, level of GSH in noninfected rats in different days was not significantly changed while, in infected rats on the 7th day after infection, GSH was significantly increased comparing to 5th (*p* = 0.002), 10th (*p* = 0.001), and 45th (*p* = 0.001) day after infection. After comparing GSH level in infected and uninfected rats on the days 0 (*p* = 0.57), 5 (*p* = 0.22), and 45 (*p* = 0.55) after infection, significant changes were not found whereas on day 7 (*p* = 0.019) GSH level was substantially increased (Table 2).

Changes of GPX activity on days 5 (*p* = 0.03) and 7 (*p* = 0.04) after infection were significant in infected group. Also significant differences were detected in GPX activity between the infected rats and the controls on days 5 (*p* = 0.03) and 7 (*p* = 0.02) after infection (Table 3).

As shown in Table 4 the values for the activity of catalase in infected and uninfected rats were constant throughout the experiment. Also no significant differences were detected in catalase activity between the infected rats and the controls on days 0 (*p* = 0.72), 5 (*p* = 0.57), 7 (*p* = 0.51), 10 (*p* = 0.53), and 45 (*p* = 0.75) after infection.

The control value for the total antioxidant capacity in uninfected rats was constant throughout all stages of experiment whilst a statically significant decrease in the total antioxidant capacity was noticed on day 7 after infection in infected ones (*p* = 0.004). Significant difference was detected in total antioxidant capacity between the infected rats and the control ones on day 7 after infection (*p* = 0.02) (Table 5).

Changes of MDA level in different days in infected (*p* = 0.57) and uninfected rats (*p* = 0.21) were not significant. Also after comparing MDA level in infected and uninfected groups on days 0 (*p* = 0.53), 5 (*p* = 0.49), 7 (*p* = 0.12), 10 (*p* = 0.15), and 45 (*p* = 0.28) after infection, significant changes were not found (Table 6).

## 4. Discussion

A critical aspect of host defense to* Toxoplasma gondii* is the activation of parasiticidal mechanisms in host leukocytes. Mononuclear phagocytes, particularly macrophages that have been activated by lymphokines, are the principal defense against intracellular pathogens such as* T. gondii* and respiratory burst (rapid release of reactive oxygen species) plays an important role against* T. gondii* [18]. One of the mechanisms that protect the host cells against excess free radicals is the enzymatic antioxidant defense which includes SOD, catalase, peroxiredoxins, flavor hemoglobins, and glutathione S-transferase/GPX coupled to glutathione reductase. SOD is a key enzyme that appears to act as the first line defense against ROS but in the present study its activity was constant throughout all stages of experiment in infected rats.

Glutathione peroxidase and catalase are the two main enzymes involved in H_2_O_2_ detoxification [19]. Hydrogen peroxide (H_2_O_2_) is one of the main reactive oxygen species (ROS) leading to oxidative stress [20]. H_2_O_2_ is continuously generated by several enzymes (including superoxide dismutase, glucose oxidase, and monoamine oxidase) and must be degraded to prevent oxidative damage. The cytotoxic effect of H_2_O_2_ is thought to be caused by hydroxyl radicals generated from iron-catalyzed reactions, causing subsequent damage to DNA, proteins, and membrane lipids [21]. According to our results significant changes of catalase activity were not found during the experiment in infected rats while GSH level and GPX activity were substantially increased on the 7th day after infection. This is despite the fact that GSH elevation may be due to stimulation of antioxidant defense system against infection. GPX are a family of selenium-containing antioxidant enzymes that catalyze the reduction of hydrogen peroxide in the presence of reduced glutathione [22]. Glutathione exists in both reduced (GSH) and oxidized (GSSG) states. In the reduced state, the thiol group of cysteine is able to donate a reducing equivalent to unstable molecules such as reactive oxygen species. In donating an electron, glutathione itself becomes reactive but readily reacts with another reactive glutathione to form glutathione disulfide (GSSG). GSH can be regenerated from GSSG by the enzyme glutathione reductase (GSR) [23].

For reduction of glutathione via glutathione reductase, which converts reactive H_2_O_2_ into H_2_O by glutathione peroxidase, NADPH is necessary. One of the most important metabolic pathways that generate NADPH is pentose phosphate pathway. It is possible that pentose phosphate pathway or other NADPH-generation pathways are increased in response to oxidative stress in acute toxoplasmosis (on day 7 after infection). However more detailed studies should be done to prove this claim. Based on the results of present study statistically significant decrease in the total antioxidant capacity was noticed on day 7 after infection in infected rats and significant difference was detected in total antioxidant capacity between the infected rats and the control ones on day 7 after infection. Total antioxidant capacity is based on the cumulative action of all the enzymatic (like SOD, catalase, GPX, etc.) and nonenzymatic antioxidants (like vitamins E and C, glutathione, melatonin, etc.) present in plasma and body fluids, thus providing an integrated parameter rather than the simple sum of measurable antioxidants.

Measuring serum TAC may help in the evaluation of physiological, environmental, and nutritional factors of the redox status in humans. Determining serum TAC may help to identify conditions affecting oxidative status* in vivo*. The measure of TAC considers the cumulative action of all the antioxidants present in serum and body fluids, thus providing an integrated parameter rather than the simple sum of measurable antioxidants. The capacity of known and unknown antioxidants and their synergistic interaction is therefore assessed, thus giving an insight into the delicate balance* in vivo* between oxidants and antioxidants [24]. In the present study GSH level was increased on day 7 after infection but TAC was decreased. Probably other antioxidants have been consumed to compensate and cope with oxidative stress and to prevent injuries from stress and effective infection control. Reduction of TAC is due to oxidative stress and returning to the basic state is probably due to the compatibility with stress. The enhanced production of ROS during infection can pose a threat to biomolecules by oxidation of proteins, damage to nucleic acids, and causing peroxidation of lipids [25]. MDA is a highly reactive aldehyde compound that results from lipid peroxidation of polyunsaturated fatty acids. The production of this aldehyde is used as a biomarker of oxidative stress [26]. In the present study MDA level was increased on day 7 after infection but it was not significant. Overall it seems that on day 7  after infection oxidative stress has occurred but it was not terminated to end product of lipid peroxidation elevation. Engin et al. have suggested that in toxoplasmosis MDA elevation is restricted to intracellular area and serum MDA level does not change [27].

In another point of view some studies have shown that inhibitors of mitochondrial function and inducers of oxidative stress can induce* Toxoplasma* encystment* in vitro* [28, 29]. Based on the present study perhaps antioxidant defense system is one of the effective mechanisms in tachyzoite-bradyzoite interconversion. However this study alone cannot confirm the above hypothesis. Understanding tachyzoite-bradyzoite interconversion process could help in designing new chemotherapeutic agents capable of eliminating tissue cysts. Therefore, further studies are required in this issue.

## Figures and Tables

**Table 1:** Mean ± standard error of SOD activity (U/mL) in noninfected and *T. gondii* infected rats.

	Days after infection				
	0	5	7	10	45
Infected	98.2 ± 10.3^Aa^	103.1 ± 9.3^Aa^	114.2 ± 13.9^Aa^	114.3 ± 15.3^Aa^	104.6 ± 17.8^Aa^
Noninfected	94.6 ± 8.7^Aa^	85.1 ± 16.3^Aa^	107.2 ± 16.2^Aa^	91.1 ± 18.05^Aa^	110 ± 10.8^Aa^

Values in columns and rows with different uppercase and lowercase superscripts are significantly different (*p* < 0.05).

**Table 2:** Mean ± standard error of GSH level (*μ*mol/mL) in noninfected and *T. gondii* infected rats.

	Days after infection				
	0	5	7	10	45
Infected	72.6 ± 5.3^Aa^	78.6 ± 7.7^Aa^	174.7 ± 16.4^Bb^	52.1 ± 6.3^Aa^	75.4 ± 18.3^Aa^
Noninfected	70.3 ± 5.4^Aa^	66.6 ± 4.9^Aa^	98.4 ± 14.9^Bb^	106.2 ± 20.4^Aa^	90.4 ± 14.4^Aa^

Values in columns and rows with different uppercase and lowercase superscripts are significantly different (*p* < 0.05).

**Table 3:** Mean ± standard error of GPX activity (U/mL) in noninfected and *T. gondii* infected rats.

	Days after infection				
	0	5	7	10	45
Infected	114.4 ± 11.6^Aa^	313.5 ± 43.53^Bb^	296.47 ± 42.1^Bb^	128.1 ± 30.3^Aa^	118.6 ± 28.7^Aa^
Noninfected	117.3 ± 10.9^Aa^	114.6 ± 14.4^Aa^	107.4 ± 19.4^Aa^	121.6 ± 23.4^Aa^	107.1 ± 18.1^Aa^

Values in columns and rows with different uppercase and lowercase superscripts are significantly different (*p* < 0.05).

**Table 4:** Mean ± standard error of catalase activity (U/mL) in noninfected and *T. gondii* infected rats.

	Days after infection				
	0	5	7	10	45
Infected	513.5 ± 82.3^Aa^	466.8 ± 88.1^Aa^	515.8 ± 63.2^Aa^	551.5 ± 32.4^Aa^	408.5 ± 78.8^Aa^
Noninfected	532.5 ± 78.2^Aa^	528.5 ± 75.6^Aa^	427.5 ± 105.3^Aa^	471.2 ± 88.7^Aa^	449.7 ± 95.3^Aa^

Values in columns and rows with different uppercase and lowercase superscripts are significantly different (*p* < 0.05).

**Table 5:** Mean ± standard error of total antioxidant capacity (mmol/L) in noninfected and *T. gondii* infected rats.

	Days after infection				
	0	5	7	10	45
Infected	987.2 ± 218.3^Aa^	1016 ± 138.5^Aa^	366.3 ± 53.6^Bb^	1064.0 ± 70.7^Aa^	1124.0 ± 105.3^Aa^
Noninfected	874.1 ± 128.3^Aa^	869.6 ± 125.5^Aa^	895.6 ± 314.8^Aa^	816.1 ± 169.9^Aa^	961.72 ± 172.3^Aa^

Values in columns and rows with different uppercase and lowercase superscripts are significantly different (*p* < 0.05).

**Table 6:** Mean ± standard error of MDA (*µ*mol/L) in noninfected and *T. gondii* infected rats.

	Days after infection				
	0	5	7	10	45
Infected	20.6 ± 3.2^Aa^	22.16 ± 5.4^Aa^	13.56 ± 2.9^Aa^	13.07 ± 1.2^Aa^	15.5 ± 2.8^Aa^
Noninfected	19.6 ± 3.2^Aa^	18.2 ± 2.7^Aa^	25.6 ± 6.01^Aa^	18.5 ± 3.2^Aa^	21.7 ± 4.1^Aa^

Values in columns and rows with different uppercase and lowercase superscripts are significantly different (*p* < 0.05).
